# Correction: Isolation and Identification of *Oedogonium* Species and Strains for Biomass Applications

**DOI:** 10.1371/journal.pone.0097373

**Published:** 2014-05-05

**Authors:** The PLOS ONE Staff

## Notice of Republication

This article was republished on April 17, 2014, to correct the figure order. Please download the article again to view the correct version. The originally published, uncorrected article and the republished, corrected article are provided here for reference.

## Supporting Information

File S1 Originally published, uncorrected article.
(PDF)

File S2 Republished, corrected article.
(PDF)
