# Supplementary material for: Isolation and Identification of Oedogonium Species and Strains for Biomass Applications
Source: PLoS One. 2014 Mar 6;9(3):e90223. doi: 10.1371/journal.pone.0090223 (PMC3946159; doi:10.1371/journal.pone.0090223)
Supplement: Table S1 — PERMANOVA post hoc tests for constant temperature experiment. Results of PERMANOVA post hoc tests on main effect of Week x Isolate (We x Is) in the constant temperature experiment. P values for each test are presented, significant terms shown in bold. (DOCX) [file pone.0090223.s003.docx]

**SUPPORTING INFORMATION**

**Table S1 PERMANOVA post hoc tests for constant temperature experiment**

Results of PERMANOVA post hoc tests on main effect of Week x Isolate (We x Is) in the constant temperature experiment. *P* values for each test are presented, significant terms shown in bold.

| **Isolate** | **Weeks 1 and 2** | **Weeks 1 and 3** | **Weeks 2 and 3** |
| --- | --- | --- | --- |
| Riv1 | 0.120 | **<0.001** | **0.008** |
| Riv2 | **0.002** | 0.198 | **0.002** |
| Riv3 | **0.003** | 0.334 | **<0.001** |
| Riv4 | **0.007** | 0.734 | 0.473 |
| Riv5 | 0.148 | 0.228 | 0.799 |
| Tar1 | **0.002** | **0.035** | 0.312 |
| Tar2 | 0.332 | **<0.001** | **0.002** |
| Tar3 | 0.444 | 0.189 | **0.009** |
| Tar4 | 0.112 | 0.595 | 0.474 |
| Tsv1 | 0.221 | 0.111 | **0.021** |
| Tsv2 | **<0.001** | 0.151 | **0.001** |
