# Supplementary material for: Isolation and Identification of Oedogonium Species and Strains for Biomass Applications
Source: PLoS One. 2014 Mar 6;9(3):e90223. doi: 10.1371/journal.pone.0090223 (PMC3946159; doi:10.1371/journal.pone.0090223)
Supplement: Text S1 — Morphological characteristics of Oedogonium isolates. (DOCX) [file pone.0090223.s004.docx]

**SUPPORTING INFORMATION**

**Text S1 Morphological characteristics of *Oedogonium* isolates**

*Riv1*: Vegetative cells are straight sided cylindrical with smooth cell walls; 50-52 µm diameter, L/D 3-8. Lions claw feet basal cell structure. Female and male structures not observable.

*Riv2*: Vegetative cells are short and cylindrical with a smooth cell wall; L/D 2-2.5. Spherical oogonium with capped pore opening. Oogonium opening position is supramedian to superior. Smooth sculpture on oospore. Solitary oogonia in sequence. Filamentous males. Groups of 3 to 4 antheridial cells developing in separate filaments supported by vegetative cells. Small to moderate slit/pore opening. Basal cell structured as vegetative cells. Slightly peaked dome terminal cell/cap structure.

*Riv3*: Vegetative cells are slightly capitellate with smooth and thick cell walls, 19-24 µm diameter; L/D≥3. Ovoid shaped oogonium. Lions claw feet basal cell structure. Rounded dome terminal cell/cap structure. Male structures not observable.

*Riv4*: Vegetative cells are cylindrical with smooth cell walls; L/D 2.5c. Elliptical oogonium with circumcision opening. Oogonium opening position is supreme. No sculpture on oospore. Foot domed basal cell structure. Dwarf males. Antheridial cells develop inside body cell, one body cell to 2 antheridia, supported by vegetative cells. Rounded terminal cell/cap structure.

*Riv5*: Vegetative cells are 5 times undulate with smooth layered cell walls, 18-26µm diameter; L/D 2-3. Spherical oogonium with "pore and circumcision" opening. Oogonium opening position is median or just below. Laminated sculpture on oospore with a smooth layered outer wall. Single or paired oogonia in sequence. Dwarf males. Two antheridial cells developing in the body cell of the dwarf male supported by vegetative cells. Wide or torn slit/pore opening. Basal cell structure with claws. Domed or layered terminal cell/cap structure.

*Tar1:* Vegetative cells are cylindrical with smooth cell walls; 22-24µm diameter L/D 1.5-4. Globose shaped oogonium with length of 34µm and diameter of 36µm. Oogonium opening position is superior, opening shape is obscure but possibly a pore. Basal part of oogonium is flat. Appears to be single number of oogonia in sequence. Vegetative supporting cells. Wide slit/pore opening. Male structures not observable.

*Tar2:* Vegetative cells are cylindrical with slight expansion at the top and with smooth cell walls; c.12µm diameter. Pyriform shaped oogonium, with a circumcision shaped opening. Basal part of oogonium is flat. Single oogonia in sequence. Vegetative supporting cells. Domed terminal cell/cap structure. Male structures not observed.

*Tar3:* Vegetative cells are cylindrical to very slightly conical with smooth cell wall, 10-12µm diameter. Barrel shaped oogonium. Basal part of oogonium is flat. Appears to be single number of oogonia in sequence. Male structures not observable.

*Tar4:* Vegetative cells are cylindrical with smooth cell walls; 20-28µm diameter, L/D 1.75-3. Barrel shaped oogonium with pore shaped opening. Oogonium opening position is superior. Basal part of oogonium is flat. Appears to be single number of oogonia in sequence. Vegetative supporting cells. Wide slit/pore opening. Male structures not observable.

*Tsv1*: Thick walled cylindrical vegetative cells, 26-29 µm diameter, L/D 2-2.5. Slight ovoid "resting cells". Smooth opening on oogonium. Rounded dome terminal cell/cap structure.

*Tsv2*: Often contains regular runs of similar sized cylindrical cells, L/D 1-2. Vegetative cells are straight sided cylinders with smooth cell walls; diameter 26-31 µm, L/D 1.5-2.
