# Supplementary material for: Correction: Isolation and Identification of Oedogonium Species and Strains for Biomass Applications
Source: PLoS One. 2014 May 5;9(5):e97373. doi: 10.1371/journal.pone.0097373 (PMC4010544; doi:10.1371/journal.pone.0097373)
Supplement: File S1 — Originally published, uncorrected article. (PDF) [file pone.0097373.s001.pdf]

OPEN ACCESS Freely available online

PLOS | ONE

Isolation and Identification of *Oedogonium* Species and Strains for Biomass Applications

Rebecca J. Lawton[1]*, Rocky de Nys[1], Stephen Skinner[2], Nicholas A. Paul[1]

1 School of Marine and Tropical Biology, James Cook University, Townsville, Queensland, Australia, **2** Molonglo Catchment Group, Fyshwick, Australia

Abstract

Freshwater macroalgae from the genus *Oedogonium* have recently been targeted for biomass applications; however, strains of *Oedogonium* for domestication have not yet been identified. Therefore, the objective of this study was to compare the performance of isolates of *Oedogonium* collected from multiple geographic locations under varying environmental conditions. We collected and identified wild-type isolates of *Oedogonium* from three geographic locations in Eastern Australia, then measured the growth of these isolates under a range of temperature treatments corresponding to ambient conditions in each geographic location. Our sampling identified 11 isolates of *Oedogonium* that could be successfully maintained under culture conditions. It was not possible to identify most isolates to species level using DNA barcoding techniques or taxonomic keys. However, there were considerable genetic and morphological differences between isolates, strongly supporting each being an identifiable species. Specific growth rates of species were high (>26% day^{-1}) under 7 of the 9 temperature treatments (average tested temperature range: 20.9–27.7°C). However, the variable growth rates of species under lower temperature treatments demonstrated that some were better able to tolerate lower temperatures. There was evidence for local adaptation under lower temperature treatments (winter conditions), but not under higher temperature treatments (summer conditions). The high growth rates we recorded across multiple temperature treatments for the majority of species confirm the suitability of this diverse genus for biomass applications and the domestication of *Oedogonium*.

Citation: Lawton RJ, de Nys R, Skinner S, Paul NA (2014) Isolation and Identification of *Oedogonium* Species and Strains for Biomass Applications. PLoS ONE 9(3): e90223. doi:10.1371/journal.pone.0090223

Editor: Jonathan H. Badger, J. Craig Venter Institute, United States of America

Received September 10, 2013; **Accepted** January 30, 2014; **Published** March 6, 2014

Copyright: © 2014 Lawton et al. This is an open-access article distributed under the terms of the Creative Commons Attribution License, which permits unrestricted use, distribution, and reproduction in any medium, provided the original author and source are credited.

Funding: This research is supported by the Australian Government through the Australian Renewable Energy Agency and the Advanced Manufacturing Cooperative Research Centre (AMCRC), funded through the Australian Government's Cooperative Research Centre Scheme. The funders had no role in study design, data collection and analysis, decision to publish, or preparation of the manuscript.

Competing Interests: Author Stephen Skinner as he is employed by the commercial company Molonglo Catchment Group. This does not alter our adherence to all the PLOS ONE policies on sharing data and materials.

* E-mail: rebecca.lawton@jcu.edu.au

Introduction

Freshwater macroalgae have diverse applications as targets for biofuels [1], the bioremediation of waste waters [2–4], fertiliser and soil conditioners [5]and as a tool for carbon sequestration [1]. But despite their potential for biomass applications, freshwater macroalgae have thus far been under-utilised as a feedstock. To date, only a single study has compared the performance of freshwater macroalgae in order to identify target species for biomass applications. Lawton et al. [6] identified the cosmopolitan genus *Oedogonium* as a target for biomass applications due to its high productivity, favourable biochemical composition, cosmopolitan distribution and competitive dominance over other algal species. However, these findings were based on the performance of a single wild-type strain of *Oedogonium*. In order to realise the full potential of this alga for biomass applications, high productivity strains of *Oedogonium* need to be identified and domesticated.

Domestication and selective breeding of agricultural food crops has resulted in large gains in productivity compared to wild-type varieties and generated the high yielding strains which are cultivated today [7].For example, worldwide production of cereals more than doubled between 1960 and 2000 as a result of selective breeding and optimisation of cultivation techniques [8].Applying the same processes and principles used in past agricultural domestications to new target species, biofuel crops for example, will enable efficient identification of strains for domestication [9]. As a first step towards domestication, the natural variability of wild-type strains for production related traits needs to be determined and the degree to which different genotypes perform best under different environments assessed [9]. Performance tests of different provenances and strains over a range of conditions are necessary in order to reveal possible genotype by environment (G X E) interactions and determine appropriate strains to domesticate [10]. If there are strong genotype by environment interactions, some strains may be adapted to local conditions at their collection locations and may not perform well under other conditions [11]. Therefore, the best candidates for domestication of freshwater macroalgae should be strains with naturally high productivities under a range of conditions, particularly if this productivity has a genetic rather than environmental basis.

A wide range of environmental conditions can also influence the growth and productivity of macroalgae [12]. Conditions such as nutrient availability and water flow rate can be easily manipulated in intensive large scale cultivation systems; however, it is often difficult and costly to manipulate temperature. Therefore, the effect of temperature on productivity should be a key consideration when selecting strains for domestication. In general, photosynthesis and growth rates of macroalgae, and therefore productivity, increase

Table 1. Sample information.

Isolate	Date	Location	Accession number[2]	Provisional species identification	Rationale
Riv1	13/11/2012	Yass, NSW ,34°30′08″S, 146°13′56″E	KF606971	*Oedognoium* sp., belonging to capillare or crassum group.	Not possible to assign a species name based on morphological characteristics. ITS sequence does not form a clade with any other *Oedogonium* species.
Riv2	13/11/2012	Yenda, NSW,34°15′05″S, 146°12′54″E	KF606972	*Oedogonium* sp.aff.*pringsheimii Cramer* ex.*Hisn*	Identification based on morphological characteristics. Doe0 snot show the distinctly narrower males of *O. pringsheimii*. ITS sequence does not form a clade with any other *Oedogonium* species.
Riv3	14/11/2012	Jerilderie, NSW,35°21′16″S, 145°43′29″E	U/R	*Oedogonium* sp.	Not possible to assign a species name based on morphological characteristics. Could not obtain readable ITS sequence.
Riv4	14/11/2012	Rutherglen, VIC, 36°02′15″S, 146°23′41″E	KF606973	*Oedogonium implexum*	Identification based on morphological characteristics. ITS sequence does not form a clade with any other *Oedogonium* species.
Riv5	14/11/2012	Chiltern, VIC, 36°09′28″S, 146°34′36″E	U/R	*Oedogonium undulatum* var *Wissmanii*	Identification based on morphological characteristics. Could not obtain readable ITS sequence.
Tar1	31/05/2012	Tarong, QLD, 26°46′02″S, 151°55′26″E	KF606974	*Oedogonium* sp.	Not possible to assign a species name based on morphological characteristics. ITS sequence does not form a clade with any other *Oedogonium* species.
Tar2	11/10/2012	Tarong, QLD, 26°46′01″S, 151°54′56″E	KF606975	*Oedogonium* sp.	Not possible to assign a species name based on morphological characteristics. ITS sequence does not form a clade with any other *Oedogonium* species.
Tar3	11/10/2012	Tarong, QLD, 26°46′01″S, 151°54′56″E	U/R	*Oedogonium* sp.	Not possible to assign a species name based on morphological characteristics. Could not obtain readable ITS sequence.
Tar4	11/10/2012	Tarong, QLD, 26°46′01″S, 151°55′12″E	KF606976	*Oedogonium* sp.	Not possible to assign a species name based on morphological characteristics. ITS sequence does not form a clade with any other *Oedogonium* species.
Tsv1	-[1]	Townsville, QLD, 19°19′45″S, 146°45′41″E	KC701473	*Oedogonium* sp.	Not possible to assign a species name based on morphological characteristics. ITS sequence does not form a clade with any other *Oedogonium* species.
Tsv2	15/07/2012	Townsville, QLD, 19°19′57″S, 146°45′33″E	KF606977	*Oedogonium* cf *intermedium*	Most species defining morphological characteristics were not present. ITS sequence does not form a clade with any other *Oedogonium* species.

[1]This strain has been maintained in continuous culture at James Cook University for >2 years.
[2]U/R - unreadable sequence.
List of isolates used in this study, collection date and location, GenBank accession number for ITS sequences and provisional species identification based on morphological characteristics and phylogenetic trees constructed using ITS sequence data.
doi:10.1371/journal.pone.0090223.t001

with increasing temperature up to an optimum point and then rapidly decline near an upper critical temperature [13,14]. The exact parameters of these curves and temperature optima vary both between and within species; however, maximal growth rates and tolerance to temperature variation are typically correlated with the temperature regime in the local habitat of an algal species [12,13]. Consequently, strains of the same algal species inhabiting different environments may have very different optimum temperatures for growth and tolerances to temperature variation.

The objective of the current study was to compare the performance of isolates of *Oedogonium* collected from multiple geographic locations under varying environmental conditions. (We use the term isolates here to refer to individual samples or variants within a species, including strains, ecotypes or genotypes).Our specific aims were to 1) collect and identify wild-type isolates of *Oedogonium* from three geographic locations; and 2) measure the growth of these isolates under a range of temperature treatments corresponding to ambient conditions in each geographic location. Ideally, we wanted to identify isolates that exhibit naturally high growth rates under a range of temperature conditions. Assessing the performance of a variety of *Oedogonium* isolates collected from a range of environments will also provide insights as to whether the high growth rates of *Oedogonium* recorded by Lawton et al. [6] are unique to the single wild-type isolate tested in their study or are characteristic of the genus.

Methods

Sample collection and isolation

Samples of freshwater macroalgae were collected from naturally occurring water bodies, irrigation channels and wetland areas in three distinct geographic regions of Australia– Riverina (35°S,

145°E), Tarong (26°S, 151°E) and Townsville (19°S, 146°E).Permission was obtained from owners and local authorities where appropriate to collect samples. These locations were chosen as they encompass a broad range of environmental conditions (Supporting information, Fig. S1) and they are focal areas for a range of industries with potential biomass applications for freshwater algae. The Riverina region has a cool temperate climate and is a centre for agricultural production; Tarong has a warm temperate climate and is the location of a coal fired power station which produces large amounts of complex industrial waste effluents; and Townsville has a tropical climate and is a centre for biofuels research. Twenty samples were collected from the Riverina, 5 samples from Tarong and 3 samples from Townsville (including Tsv1, the original isolate tested by Lawton et al. [6]). Samples were transported in water taken at the collection site back to James Cook University, Townsville, where they were identified to genus using a compound microscope. Any samples containing *Oedogonium* were maintained in nutrient enriched autoclaved freshwater in a temperature and light controlled laboratory (12:12 light:dark cycle, 50 µmol photons m^{-2} s^{-1}, 23°C), all other samples were discarded. Stock cultures of *Oedogonium* isolates were created by isolating individual filaments of *Oedogonium* from each sample and maintaining these in sterile petri dishes with nutrient enriched (Guillards F/2 medium; 12.3 mg L^{-1} nitrogen, 1.12 mg L^{-1}phosphorus) autoclaved freshwater in culture cabinets at 24.5°C with 12 hour light: 12 hour dark cycles and a light level of 50 µmol m^{-2} s^{-1}. These conditions correspond to the middle temperature treatment (Tarong) of the constant temperature experiment (see below). In some cases multiple isolates of *Oedogonium* were isolated from the same sample. In total, 26 isolates of *Oedogonium* were isolated, of which 11 were successfully scaled up into stock cultures – 5 isolates from Riverina, 4 isolates from Tarong and 2 isolates from Townsville (Table 1). Stock cultures of each isolate were maintained for at least 2 months prior to the start of each experiment to allow acclimation to the culture system and ensure that all algae were pre-exposed to identical conditions.

Isolation and identification

Isolates of *Oedogonium* were identified using DNA barcoding and taxonomic keys. Samples of each isolate were examined under dissecting and compound light microscopes and their morphological characteristics were recorded. Where possible, each sample was identified to species using taxonomic keys [15]. For the DNA barcoding, DNA sequences from the internal transcribed spacer (ITS) region of the ribosomal cistron were used to assign species names to isolates of *Oedogonium*. This marker has been widely used in species level phylogenetic studies of green algae and was the marker of choice in a recent phylogenetic study of *Oedogonium* [16].Genomic DNA was isolated from fresh tissue samples of each isolate using a Qiagen DNEasy Plant Mini Kit following the manufacturer's instructions. The ITS region was amplified using the primers ITS1 [17] and G4 [18]. Polymerase chain reaction (PCR) amplifications were performed in a 25 µL reaction mixture containing 1.5 U of MyTaq HS DNA polymerase (Bioline), 5× MyTaq reaction buffer, 0.4 µM each primer, and 1 µL of genomic DNA (25–30 ng). Amplifications were performed on a BioRad C1000 Thermal Cycler with a touchdown PCR cycling profile (cycling parameters: 5 min at 94°C, 30 cycles of 30 s denaturing at 95°C, 45 s annealing at 56°C with the annealing temperature decreasing by 0.5°C each cycle, 60 s extension at 72°C, and a final extension at 72°C for 5 min). PCR products were column purified using Sephadex G25 resin and sequenced in both directions by the Australian Genome Research Facility (Brisbane, Australia). If sequences were unreadable or contaminated a second PCR attempt was made and sequenced. If these sequences were also unreadable or contaminated, then DNA was re-extracted from a fresh sample and further PCRs and sequencing attempts were made. However, despite these steps we were not able to obtain readable sequences for three isolates – Riv3, Riv5 and Tar3. Sequences were edited using Bioedit [19] and submitted to GenBank under the accession numbers given in Table 1.

Isolates were identified based on their DNA sequences by constructing phylogenetic trees using sequences downloaded from GenBank. All publically available *Oedogonium* ITS sequences were downloaded. Duplicate sequences were removed from each dataset and then all remaining sequences were aligned with ours and trimmed to a standard length in MEGA 5.0 [20]. The ITS dataset included 32 sequences, 24 of which were retrieved from GenBank and the alignment consisted of 766 positions. Maximum likelihood (ML) phylogenetic trees were constructed in MEGA using a *Bulbochaete rectangularis* sequence (AY962677) as an outgroup. jModelTest 2.1 [21,22]showed that the SYM+I+G model of molecular evolution best fitted the data. However, as this model was not available in MEGA we used the simple Kimura two-parameter model to estimate genetic distance [23] as this is the standard model of molecular evolution used in barcoding studies [24]. The reliability of tree topologies was estimated using bootstrapping (1,000 replicates). Pairwise differences between all sequences used in the analysis were generated in MEGA using a maximum composite likelihood model.

Constant temperature experiments

To determine which isolates of *Oedogonium* would be suitable for targets for biomass applications, growth trials were conducted on all isolates under three temperature treatments that were kept constant at all times. Nine filaments of each isolate were cut to a standardised length of 6 mm. Three filaments from each isolate were then grown under each of three constant temperature treatments (21.3°C, 24.5°C, and 27.7°C) in culture cabinets (Sanyo MLR-351) with 12 hour light: 12 hour dark cycles and a light level of 50 µmol m^{-2} s^{-1} (Philips TLD 36W/850 Daylight) for 7 days. These temperatures correspond to the mean annual 3pm air temperature in Wagga Wagga – the central point of our Riverina sampling locations (21.3°C), Kingaroy – the closest weather station to Tarong (24.5°C) and Townsville (27.7°C) (Table 2).This range of temperatures also represents the lower range of Townsville (21.3°C) and the upper range of Wagga Wagga and Kingaroy (27.7°C). Each individual filament was maintained in a sterile 60 mm petri dish with nutrient enriched autoclaved freshwater and photographed under a dissecting microscope (Olympus model SZ61) at the start and end of the 7 day period (Supporting information, Fig. S2). The 2-dimensional surface area of filaments was determined using ImageJ [25]. Specific growth rates were calculated for each individual replicate of each isolate using the equation SGR (% day^{-1}) = $Ln(B_f/B_i)/T{*}100$, where B_f and B_i are the final and initial surface areas (mm^2) and T is the number of days in culture. The experiment was repeated a further two times using new filaments from stock cultures at the start of each 7 day period to give a total of 3 replicate weeks of data. Permutational analysis of variance (PERMANOVA) was used to analyse the effects of isolate, temperature (both fixed effects) and week (random effect) on the specific growth rate of isolates. Analyses were conducted in Primer v6 (Primer-E Ltd, UK) using Bray-Curtis dissimilarities on fourth root transformed data and 9999 unrestricted permutations of raw data [26].

Table 2. Temperature treatments for growth experiments.

		Constant	Summer variable			Winter variable		
**Region**	**Weather station**		**Min**	**Average**	**Max**	**Min**	**Average**	**Max**
Riverina	Wagga Wagga AMO	21.3	16.3	20.9	32.2	2.9	5.8	12.9
Tarong	Kingaroy airport	24.5	18.0	21.8	30.9	3.4	8.1	19.4
Townsville	Townsville aero	27.7	24.7	26.7	31.7	14.0	17.3	25.3

Temperature treatments (°C) used in growth experiments. Temperatures for each experiment were based on those recorded by the Australian Bureau of Meteorology between 1981 and 2010 at the following weather stations: Wagga Wagga AMO for the Riverina region (35.16°S, 147.46°E), Kingaroy airport for the Tarong region (26.57°S, 151.84°E) and Townsville aero for the Townsville region (19.25°S, 146.77°E). Treatments for the constant temperature experiment correspond to the mean annual 3pm temperature in each location. Treatments for the summer variable temperature experiment correspond to the minimum and maximum mean temperatures recorded in January for each location. Treatments for the winter variable temperature experiment correspond to the minimum and maximum mean temperatures recorded in July for each location. Average daily values for each temperature profile in the variable temperature experiments are given. See methods and Figures 1B and 2B for more detail on the variable temperature treatments.
doi:10.1371/journal.pone.0090223.t002

Variable temperature experiments

To provide further insights into the performance of isolates under different temperature conditions, growth trials were also conducted on isolates using a summer variable temperature treatment and a winter variable temperature treatment. In these variable temperature experiments, temperature treatments increased and decreased during the day to represent natural variations recorded in summer (January) and winter (July) at each location (Table 2; Figs. 1B and 2B).Temperatures were maintained at the minimum summer or winter temperature from 6 pm until 6 am, then increased in equal increments every 30 minutes to reach the maximum summer or winter temperature at 12 pm. Temperatures were maintained at the maximum between 12 pm and 2 pm, then decreased in equal increments every 30 minutes from 2 pm to reach the minimum summer or winter temperature at 6pm. These profiles resulted in average daily temperatures of 20.9°C for the Riverina treatment, 21.8°C for the Tarong treatment and 26.7°C for the Townsville treatment in the summer variable temperature experiment; and 5.8°C for the Riverina treatment, 8.1°C for the Tarong treatment and 17.3°C for the Townsville treatment in the winter variable temperature experiment (Table 2). The methods used were identical to the constant temperature experiment except that each variable temperature experiment was run for a single period of 7 days. Permutational analysis of variance (PERMANOVA) was used to analyse the effects of isolate and temperature (both fixed effects) on the specific growth rate of isolates. Data for the summer variable temperature experiment and winter variable temperature experiment were analysed separately. Analyses were conducted in Primer v6 (Primer-E Ltd, UK) using Bray-Curtis dissimilarities on fourth root transformed data and 9999 unrestricted permutations of raw data [26].

Results

Strain identification

Specific morphological characteristics of species were not visible in most isolates (Supporting information, Text S1). Consequently, we were only able to assign species names to 2 of our isolates based on morphological characteristics – isolate Riv4 was identified as *Oedogonium implexum* and isolate Riv5 as *Oedogonium undulatum* var *Wissmanii* (Table 1).The DNA sequence analysis was similarly inconclusive in terms of matching isolates with extant species of *Oedogonium*. None of our isolates had identical ITS sequences to any of the *Oedogonium* sequences downloaded from GenBank. Furthermore, none of the isolates formed tight clades in the phylogenetic tree with any GenBank sequences (Fig. 3). Therefore it was not possible to assign species names to any isolates based on the ITS phylogenetic tree. Pairwise differences between GenBank samples included in the analysis ranged from 0.002 to 0.181. As the smallest pairwise distance between a sequence from our isolates and any other sequence was 0.005 (between isolates Tar4 and Tar1), it was also not possible to use pairwise distances to infer that any of our isolates were the same species as other isolates or GenBank samples included in the analysis. However, the higher pairwise differences recorded for our isolates (all >0.005), and the fact that all isolates formed distinct clades on the phylogenetic tree and did not group with any other sequences, demonstrate that isolates of *Oedogonium* are genetically distinct and therefore provides strong support that each isolate is a genetically differentiated species.

Constant temperature experiments

Average specific growth rates were high across all isolates ranging from 38.5% day^{-1} (±2.9 S.E.) at 21.3°C (Riverina treatment) to 45.3% day^{-1} (±2.3 S.E.) at 27.7°C (Townsville treatment) in the constant temperature experiment (Fig. 4). Specific growth rates varied significantly between isolates of *Oedogonium*, however this effect was not consistent between the three replicate weeks of the experiment (Table 3). There were significant differences in growth rates between weeks for all isolates except Riv5 and Tar4 ($P<0.05$) (Supporting information, Table S1). Most isolates had lower specific growth rates in week 2 of the experiment compared to week 1 and 3. There was no obvious effect of collection location on specific growth rate (Fig. 4), with isolates collected from the same location attaining their highest growth rates under different temperature treatments. For example, growth of Riverina isolate Riv2 was highest at 21.3°C (Riverina treatment), while that of Riverina isolate Riv3 was highest at 27.7°C (Townsville treatment).

Variable temperature experiments

Growth rates in the summer variable temperature experiment were similar to those seen in the constant temperature experiment. Average specific growth rates varied across all isolates, ranging from 31.9% day^{-1} (±5.4 S.E.) under the Riverina temperature treatment, to 42.3% day^{-1} (±6.8 S.E.) under the Tarong temperature treatment (Fig. 1A). However, neither isolate nor temperature had any significant effect on growth in this experiment (Table 3). In the winter variable temperature experiment, growth rates were much lower overall compared to the summer variable temperature experiment and the constant

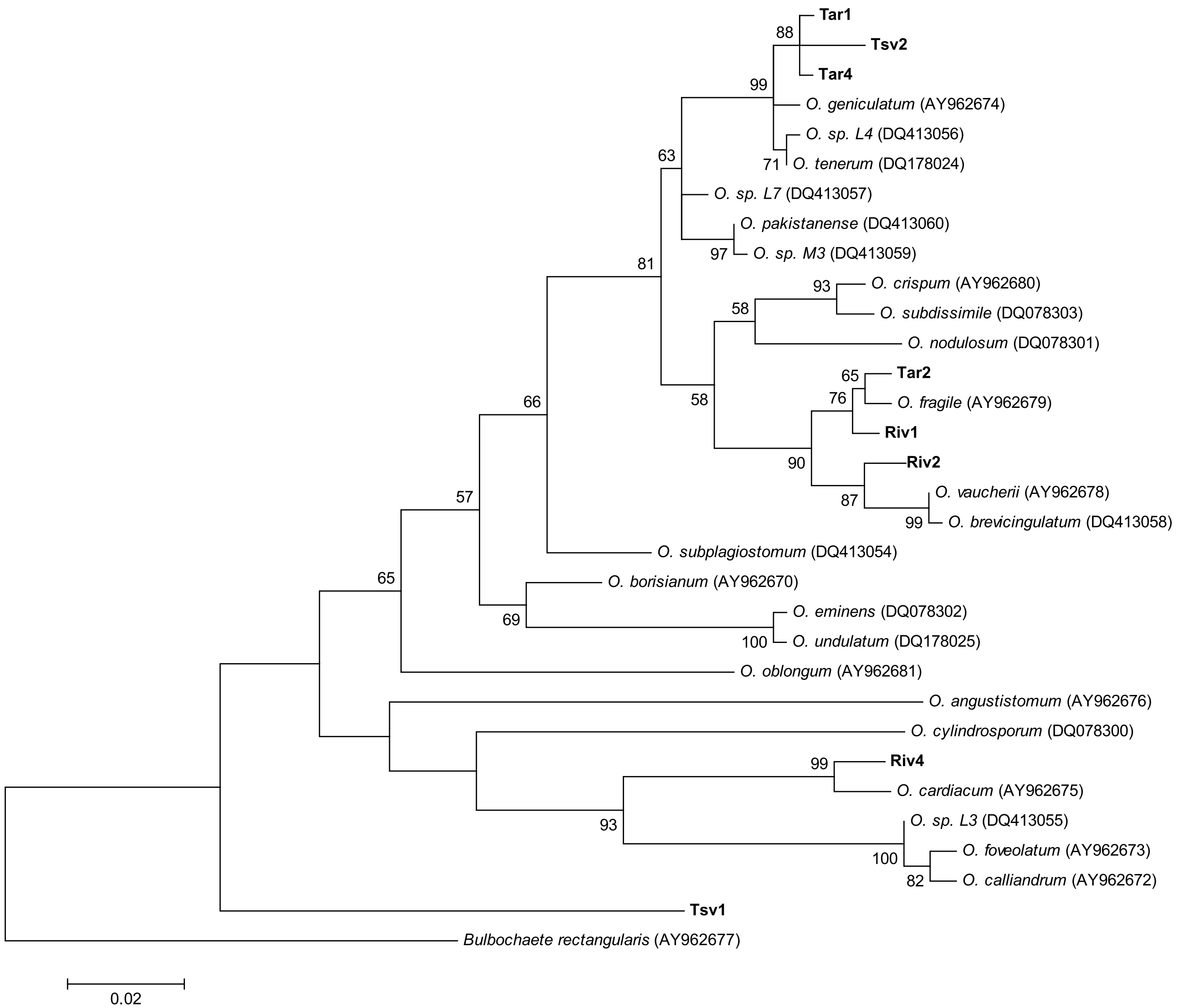

Figure 1. Growth rates of *Oedogonium* isolates and temperature profiles for the summer variable temperature experiment. A) Mean (±S.E.) specific growth rates (% day^{-1}) of isolates of *Oedogonium* collected from the Riverina (Riv), Tarong (Tar) and Townsville (Tsv) and grown under three variable temperature treatments and B)temperature profiles for Riverina, Tarong and Townsville treatments in the summer variable temperature experiment. These profiles result in average daily temperatures of 20.9°C for the Riverina treatment, 21.8°C for the Tarong treatment and 26.7°C for the Townsville treatment.
doi:10.1371/journal.pone.0090223.g001

temperature experiment. Across all isolates, average specific growth rates ranged from 2.6% day^{-1} (±2.1 S.E.) under the Riverina temperature treatment to 51.7% day^{-1} (±5.3 S.E.) under the Townsville temperature treatment (Fig. 2A). Temperature had a significant effect on the growth rate of isolates (Table 3) and growth was significantly higher under the Townsville temperature treatments compared to the Tarong and Riverina treatment ($P<0.001$, PERMANVOA post hoc tests for main effect of temperature). Specific growth rates were <10%day^{-1} for all isolates except Riv2 under the Riverina temperature treatment and <10% day^{-1} for all isolates except Riv2, Riv3 and Riv5 under the Tarong temperature treatment. In contrast, growth rates ranged from 26–85% day^{-1} for all isolates under the Townsville temperature treatment.

Discussion

Determining the natural variability of wild-type isolates for production related traits and the degree to which different isolates perform best under different environments is an essential starting point for the domestication of new target species [9]. Our survey of natural populations in three geographic regions of Australia identified 11isolates of *Oedogonium* that were successfully maintained under culture conditions. Despite being collected from a range of environments and comprising multiple species of *Oedogonium*, supported by molecular data, the majority of these isolates had high specific growth rates across a range of temperature treatments (>30% day^{-1} in the constant temperature experiment and summer variable temperature experiment). These specific growth rates are considerably higher than those recorded for marine macroalgae in similar studies [27,28]. The high

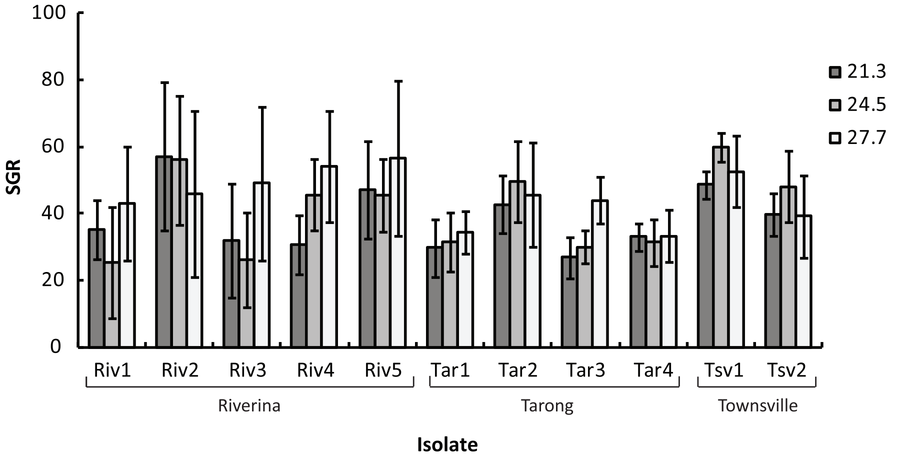

Figure 2. Growth rates of *Oedogonium* isolates and temperature profiles for the winter variable temperature experiment. A) Mean (±S.E.) specific growth rates (% day^{-1}) of isolates of *Oedogonium* collected from the Riverina (Riv), Tarong (Tar) and Townsville (Tsv) and grown under three variable temperature treatments and B)temperature profiles for Riverina, Tarong and Townsville treatments in the winter variable temperature experiment. These profiles result in average daily temperatures of 5.8°C for the Riverina treatment, 8.1°C for the Tarong treatment and 17.3°C for the Townsville treatment.
doi:10.1371/journal.pone.0090223.g002

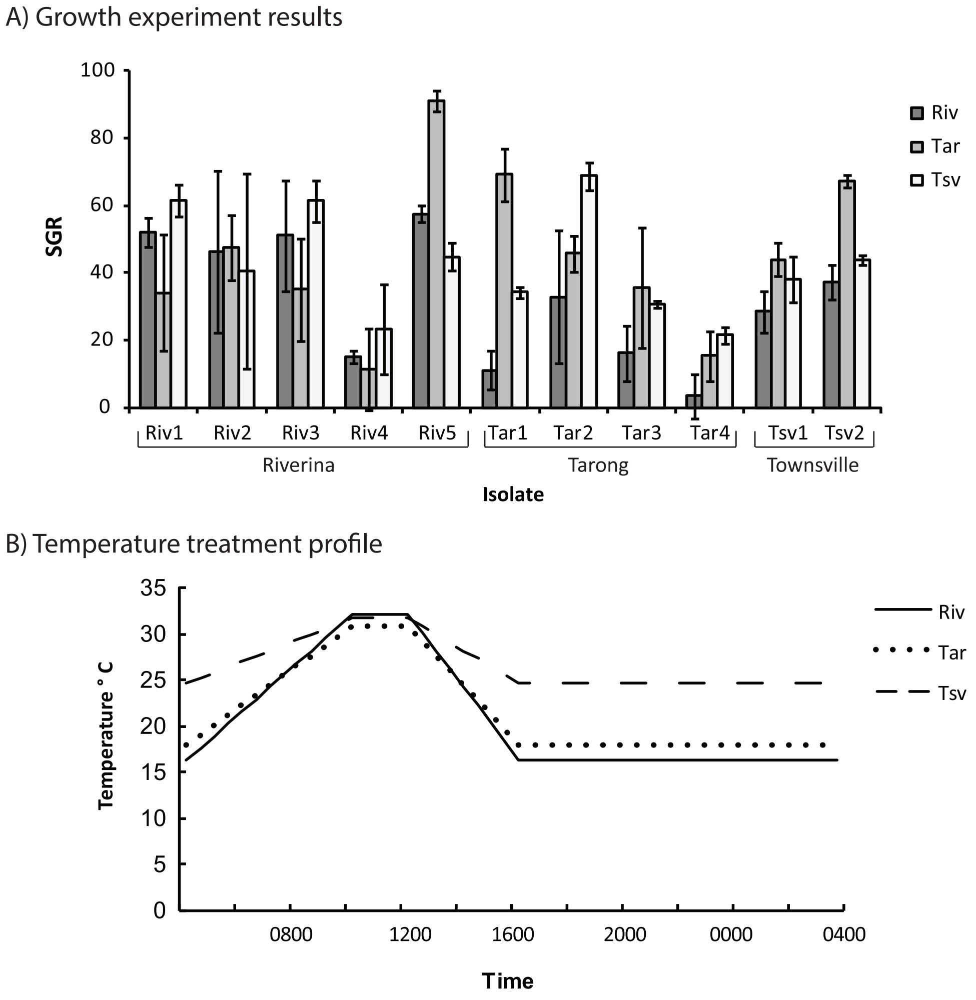

Figure 3. Maximum likelihood tree of *Oedogonium* internal transcribed spacer (ITS) sequence data. Maximum likelihood tree of *Oedogonium* internal transcribed spacer (ITS) sequence data (scale at bottom). Numbers near each node refer to bootstrap support values, nodes with <50% bootstrap support are not labelled. Isolates collected in this study shown in bold. Numbers accompanying the species names are GenBank accession numbers for the sequences used in the analysis.
doi:10.1371/journal.pone.0090223.g003

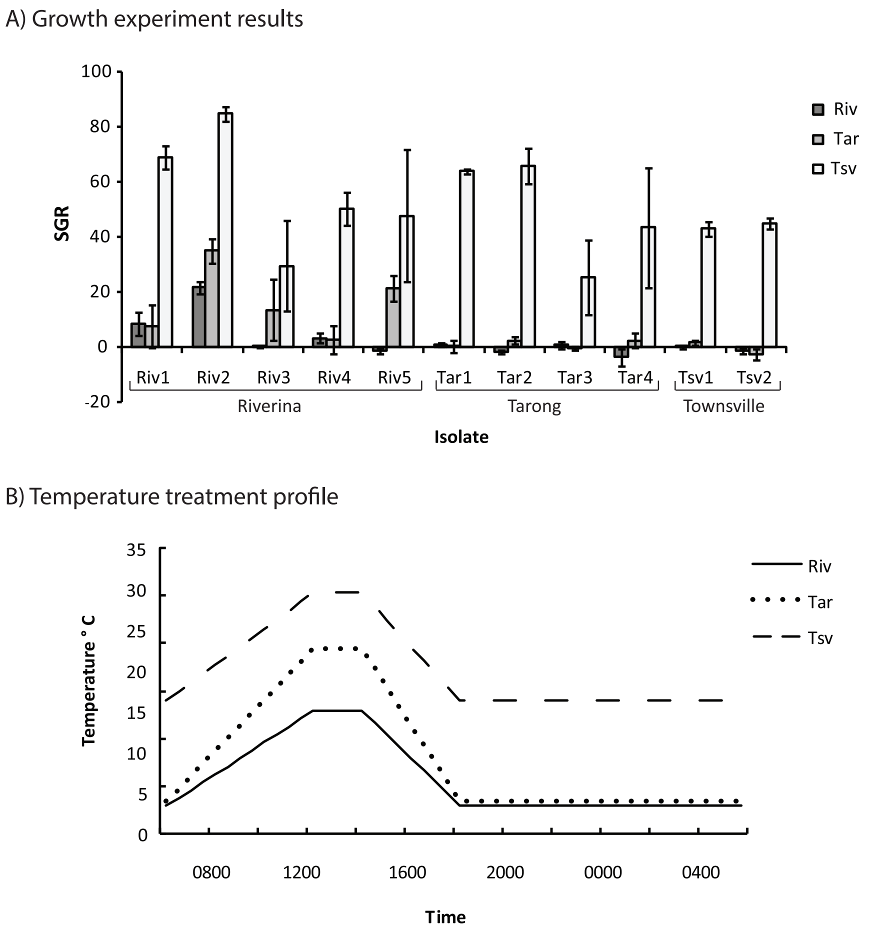

Figure 4. Growth rates of *Oedogonium* isolates for the constant temperature experiment. Mean (±S.E.) specific growth rates (% day^{-1}) of isolates of *Oedogonium* collected from three locations (Riverina, Tarong, Townsville) and grown under three constant temperature treatments. Temperatures are average daily temperatures for each profile (°C).
doi:10.1371/journal.pone.0090223.g004

Table 3. Results of permutational analyses of variance (PERMANOVAs) testing the effects of temperature (Te),isolate(Is) and week (We)on specific growth rates of *Oedogonium* in the constant temperature experiment; and the effects of temperature (Te) and isolate (Is)on specific growth rates of *Oedogonium* in the summer and winter variable temperature experiments.

		Constant		Summer variable		Winter variable	
**Source**	**df**	**F**	**P**	**F**	**P**	**F**	**P**
Te	2	0.71	0.575	1.89	0.159	**26.09**	**<0.001**
Is	10	1.09	0.374	1.55	0.124	1.83	0.069
We[1]	2	**24.23**	**<0.001**				
Te x Is	20	0.94	0.539	1.02	0.449	1.29	0.215
Te x We[1]	4	1.46	0.204				
Is x We[1]	20	**3.47**	**<0.001**				
Te x Is x We[1]	40	1.11	0.297				

[1]Week was not a factor in the summer and winter variable temperature experiments.
Pseudo F (F) and P values are presented, significant terms shown in bold.
doi:10.1371/journal.pone.0090223.t003

performance of the majority of isolates (species) across the range of temperature treatments tested here further confirms the findings of Lawton et al. [6] that species of *Oedogonium* are ideal targets for biomass applications and should be a focus for domestication efforts.

Temperature is a major factor controlling the growth of macroalgae [12,13,29]. However, temperature treatment only had a strong effect on *Oedogonium* growth rates in the winter variable temperature experiment. Growth rates across all treatments were lower in this experiment compared to the constant temperature experiment and the summer variable temperature experiment, and most isolates had very low growth rates (<10% day^{-1}) under the treatments with lowest temperatures (i.e. under Riverina and Tarong conditions). While the cause of these low growth rates is unknown, sub-lethal effects may have accumulated over the 7-day experimental period as a result of the low temperatures, limiting growth in some isolates. Low temperatures limit metabolic and photosynthetic rates in macroalgae as some of the enzymes involved in these processes are temperature dependent [12,14]. Low temperatures also impair the synthesis and functioning of photosynthetic pigment proteins [13,14] and damage cells in macroalgae by limiting electron transport and photon capture [12]. While it was not a goal of this study to quantify temperature thresholds for growth, it is possible that the growth rates recorded under the lowest temperature treatments

represent the response of isolates to a shock treatment or acute stress rather than to low temperatures *per se*. In the winter variable temperature experiment filaments were transferred directly from stock cultures maintained at 24.5°C to each treatment. For the Tarong and Riverina treatments, this represented an immediate temperature change of at least 5 to 11°C; however, it is notable that the daily temperature swings for both treatments are greater than this at 15–20°C. The rate of cooling is an important factor affecting plant responses to low temperatures [30] and could potentially be used as a strategy to ease domesticated cultures into winter conditions to enhance annual production yields.

Irrespective of the cause of low growth rates in the winter variable temperature experiment, our results demonstrate that some isolates are better able to tolerate lower temperatures than others. For example, under all three winter temperature treatments isolate Riv2 maintained specific growth rates >20% day^{-1}. Similarly, some isolates were more susceptible to low temperatures than others. For instance, isolate Tsv2 had negative growth rates under the Tarong and Riverina winter temperature treatments. Most macroalgae are capable of acclimating to variable temperatures to some degree; however, this ability is expected to be higher in algae native to habitats with large annual variations in temperature compared to those from habitats with more stable climates [12,13]. In agreement with these predictions, the best performing isolates (Riv2, Riv3 and Riv5) under the lower winter temperature treatments were all collected from the Riverina – the sampling location with the greatest annual variations in temperature. These results suggest that domestication efforts should further target isolates from regions with large annual variations in temperature as these isolates are likely to have broad temperature tolerances and therefore perform well across a wide range of conditions. The next steps in assessing the suitability of isolates for domestication and biomass applications should be the measurement of areal productivity in larger outdoor cultures [1,6]. Subsequent tests of other traits of interest between isolates, such as biochemical profiles for bioenergy potential [31], bioremediation ability [4] and carbon sequestration [1], can then be evaluated in the scaled biomass.

The lower growth rates recorded for most *Oedogonium* isolates under the Riverina and Tarong winter temperature treatments indicate that biomass production of *Oedogonium* is likely to be low over the winter period in these regions and other locations that experience similar temperatures. Estimating growth and expected production across multiple seasons is important when considering potential biomass applications for *Oedogonium*, particularly for locations where seasonal temperatures vary significantly, as use of data from a single season may result in erroneous estimates of annual productivity [12]. In contrast to growth rates recorded under the Riverina and Tarong treatments, *Oedogonium* growth rates were high (over 45% day^{-1}) under the Townsville treatment in the winter variable temperature experiment. These high growth rates indicate that Townsville, and other locations with similar temperature profiles, are target locations for *Oedogonium* cultivation as it will be possible to maintain high biomass productivity year round under ambient conditions in these locations using any of the domesticated strains.

Conclusion

This study compares, for the first time, the growth rates of multiple isolates of *Oedogonium* under a range of temperature regimes in order to identify isolates that exhibit naturally high growth rates under varying environmental conditions. The high growth rates recorded across multiple temperature treatments for the majority of isolates tested here confirm the suitability of this genus for biomass applications and demonstrate that most naturally occurring isolates of *Oedogonium* can be domesticated. All isolates tested here had similar growth rates to isolate Tsv1, the original isolate tested by Lawton et al. [6] which outperformed other genera of freshwater macroalgae across a range of metrics. Our results confirm that the genus *Oedogonium* contains many species that have a superior performance and provide a vital first step to determining the suitability of *Oedogonium* isolates for biomass applications prior to measuring areal productivity and assessing biochemical composition in large outdoor cultures. Worldwide production of food crops is based around a small number of target species. Similarly, production of marine macroalgae is dominated by only six genera [32]. Future production of freshwater macroalgae is also likely to follow these trends and rely on a select group of target genera. Our findings provide strong rationale for the inclusion of *Oedogonium* as a key target genus for the development of this industry worldwide. Our approach of collecting isolates of *Oedogonium* from multiple geographic locations and comparing their performance under a range of conditions provides a template that can be applied to other novel target species as a first step towards domestication.

Supporting Information

Figure S1 Annual temperature profiles for sampling locations. Average 9am monthly temperatures recorded in Wagga Wagga (Riverina region), Kingaroy (Tarong region) and Townsville. Error bars show mean maximum and mean minimum monthly temperatures. Data from the Australian Government Bureau of Meteorology.
(EPS)

Figure S2 Before/after photographs of *Oedogonium* growth. Microscope photos of single *Oedogonium* filament at the start and end of 7 day growth period in the constant temperature experiment.
(EPS)

Table S1 PERMANOVA post hoc tests for constant temperature experiment. Results of PERMANOVA post hoc tests on main effect of Week x Isolate (We x Is) in the constant temperature experiment. *P* values for each test are presented, significant terms shown in bold.
(DOCX)

Text S1 Morphological characteristics of *Oedogonium* isolates.
(DOCX)

Acknowledgments

This research is part of the MBD Energy Research and Development program for Biological Carbon Capture and Storage. We thank Maria Martinez, Amanda Ricketts and Boer Bao for assistance with experiments and Dean Jerry for advice on molecular analysis.

Author Contributions

Conceived and designed the experiments: RL NP RdN. Performed the experiments: RL SS. Analyzed the data: RL SS. Contributed reagents/materials/analysis tools: RL SS RnD NP. Wrote the paper: RL RnD NP.

References

1. Cole AJ, Mata L, Paul NA, De Nys R (In press) Using CO_2 to enhance carbon capture and biomass applications of freshwater macroalgae. GCB Bioenergy. DOI: 10.1111/gcbb.12097
2. Kebede-Westhead E, Pizarro C, Mulbry W (2006) Treatment of swine manure effluent using freshwater algae: Production, nutrient recovery, and elemental composition of algal biomass at four effluent loading rates. J Appl Phycol 18: 41–46.
3. Mulbry W, Kondrad S, Pizarro C, Kebede-Westhead E (2008) Treatment of dairy manure effluent using freshwater algae: Algal productivity and recovery of manure nutrients using pilot-scale algal turf scrubbers. Bioresour Technol 99: 8137–8142.
4. Saunders RJ, Paul NA, Hu Y, de Nys R (2012) Sustainable sources of biomass for bioremediation of heavy metals in waste water derived from coal-fired power generation. PloS one 7: e36470.
5. Bird MI, Wurster CM, de Paula Silva PH, Paul NA, de Nys R (2012) Algal biochar: effects and applications. GCB Bioenergy 4: 61–69.
6. Lawton RJ, de Nys R, Paul NA (2013) Selecting Reliable and Robust Freshwater Macroalgae for Biomass Applications. PloS one 8: e64168.
7. Evenson RE, Gollin D (2003) Assessing the Impact of the Green Revolution, 1960 to 2000. Science 300: 758–762.
8. Tilman D, Cassman KG, Matson PA, Naylor R, Polasky S (2002) Agricultural sustainability and intensive production practices. Nature 418: 671–677.
9. Yan J, Chen W, Luo F, Ma H, Meng A, et al. (2012) Variability and adaptability of *Miscanthus* species evaluated for energy crop domestication. GCB Bioenergy 4: 49–60.
10. Achten WMJ, Nielsen LR, Aerts R, Lengkeek AG, Kjær ED, et al. (2009) Towards domestication of *Jatropha curcas*. Biofuels 1: 91–107.
11. Kawecki TJ, Ebert D (2004) Conceptual issues in local adaptation. Ecol Lett 7: 1225–1241.
12. Lobban CS, Harrison P (2000) Seaweed ecology and physiology. Cambridge University Press, Cambridge. pp. 366.
13. Eggert A (2012) Seaweed Responses to Temperature. In: Wiencke C, Bischof K, editors. Seaweed Biology: Springer Berlin Heidelberg. pp. 47–66.
14. Davison IR (1991) Environmental effects on algal photosynthesis: temperature. J Phycol 27: 2–8.
15. Entwisle TJ, Skinner S, Lewis SH, Foard HJ (2007) Algae of Australia: Batrachospermales, Thoreales, Oedogoniales and Zygnemaceae. Collingwood, Australia: CSIRO PUBLISHING/Australian Biological Resources Study 200 p.
16. Mei H, Luo W, Liu G, Hu Z (2007) Phylogeny of Oedogoniales (Chlorophyceae, Chlorophyta) inferred from 18S rDNA sequences with emphasis on the relationships in the genus *Oedogonium* based on ITS-2 sequences. Plant Syst Evol 265: 179–191.
17. Bakker FT, Olsen JL, Stam WT (1995) Evolution of nuclear rDNA its sequences in the *Cladophora albida/sericea* clade (Chlorophyta) J Mol Evol 40: 640–651.
18. Harper JT, Saunders GW (2001) The application of sequences of the ribosomal cistron to the systematics and classification of the florideophyte red algae (Florideophyceae, Rhodophyta). Cah Biol Mar 42: 25–38.
19. Hall TA. (1999) BioEdit: a user-friendly biological sequence alignment editor and analysis program for Windows 95/98/NT; 1999. pp. 95–98.
20. Tamura K, Peterson D, Peterson N, Stecher G, Nei M, et al. (2011) MEGA5: molecular evolutionary genetics analysis using maximum likelihood, evolutionary distance, and maximum parsimony methods. Mol Biol Evol 28: 2731–2739.
21. Guindon S, Gascuel O (2003) A simple, fast, and accurate algorithm to estimate large phylogenies by maximum likelihood. Syst Biol 52: 696–704.
22. Darriba D, Taboada GL, Doallo R, Posada D (2012) jModelTest 2: more models, new heuristics and parallel computing. Nat Meth 9: 772–772.
23. Kimura M (1980) A simple method for estimating evolutionary rates of base substitutions through comparative studies of nucleotide sequences. J Mol Evol 16: 111–120.
24. Hebert PDN, Ratnasingham S, de Waard JR (2003) Barcoding animal life: cytochrome c oxidase subunit 1 divergences among closely related species. Proceedings of the Royal Society of London Series B: Biological Sciences 270: S96–S99.
25. Schneider CA, Rasband WS, Eliceiri KW (2012) NIH Image to ImageJ: 25 years of image analysis. Nat Meth 9: 671–675.
26. Anderson MJ, Gorley RN, Clarke KR (2008) PERMANOVA+ for PRIMER: Guide to Software and Statistical Methods. PRIMER-E, Plymouth, UK.
27. Lawton RJ, Mata L, De Nys R, Paul NA (2013) Algal bioremediation of waste waters from land-based aquaculture using *Ulva*: selecting target species and strains. PLoS ONE 8(10): e77344. doi:10.1371/journal.pone.0077344
28. de Paula Silva PH, McBride S, de Nys R, Paul NA (2008) Integrating filamentous 'green tide' algae into tropical pond-based aquaculture. Aquaculture 284: 74–80.
29. Raven JA, Geider RJ (1988) Temperature and algal growth. New Phytol 110: 441–461.
30. Minorsky PV (1989) Temperature sensing by plants: a review and hypothesis. Plant, Cell & Environment 12: 119–135.
31. Ross AB, Jones JM, Kubacki ML, Bridgeman T (2008) Classification of macroalgae as fuel and its thermochemical behaviour. Bioresour Technol 99: 6494–6504.
32. Paul NA, Tseng CK, Borowizka M (2012) Seaweed and microalgae. In: Lucas JS, Southgate PC, editors. Aquaculture: Farming aquatic animals and plants. Sussex, U.K.: Blackwell Publishing Ltd.
